# Outbreak of Human Immunodeficiency Virus Infection Among Heterosexual Persons Who Are Living Homeless and Inject Drugs — Seattle, Washington, 2018

**DOI:** 10.15585/mmwr.mm6815a2

**Published:** 2019-04-19

**Authors:** Matthew R. Golden, Richard Lechtenberg, Sara N. Glick, Julie Dombrowski, Jeff Duchin, Jennifer R. Reuer, Shireesha Dhanireddy, Santiago Neme, Susan E. Buskin

**Affiliations:** ^1^Public Health–Seattle & King County, Washington; ^2^Division of Allergy and Infectious Diseases, University of Washington, Seattle, Washington; ^3^Washington State Department of Health.

Although diagnoses of human immunodeficiency virus (HIV) infection among persons who inject drugs in the United States are declining, an HIV outbreak among such persons in rural Indiana demonstrated that population’s vulnerability to HIV infection (*1*). In August 2018, Public Health–Seattle and King County (PHSKC) identified a cluster of cases of HIV infection among persons living homeless, most of whom injected drugs. Investigation identified 14 related cases diagnosed from February to mid-November 2018 among women who inject drugs and men who have sex with women (MSW) who inject drugs and their sex partners. All 14 persons were living homeless in an approximately 3–square-mile area and were part of a cluster of 23 cases diagnosed since 2008. Twenty-seven cases of HIV infection were diagnosed among women and MSW who inject drugs in King County during January 1–November 15, 2018, a 286% increase over the seven cases diagnosed in 2017. PHSKC has alerted medical and social service providers and the public about the outbreak, expanded HIV testing among persons who inject drugs or who are living homeless, and is working to increase the availability of clinical and prevention services in the geographic area of the outbreak. This outbreak highlights the vulnerability of persons who inject drugs, particularly those who also are living homeless, to outbreaks of HIV infection, even in areas with high levels of viral suppression and large syringe services programs (SSPs).

## Investigation and Findings

Cluster cases met one or more of the following criteria: 1) HIV infection diagnosis in a woman or MSW in 2018, with partner services data indicating sex or sharing injection-drug equipment with a person in a previously identified cluster case; 2) HIV infection diagnosis in 2018 in a woman or MSW living homeless in the outbreak area; 3) molecular analysis indicating HIV infection with a strain related to those identified among persons meeting either of the first two criteria (HIV-TRACE genetic distance ≤1.5%) (*2*). Cases were excluded if molecular analysis indicated infection with an HIV strain unrelated to the cluster.

In July 2018, an MSW living homeless in north Seattle tested positive for acute HIV infection (HIV Ag/Ab positive, Geenius HIV negative, HIV RNA positive) at an emergency department (ED) after being evaluated with fever (patient 6) (Table 1). He did not report injecting drugs, but had paid a woman for sex. That woman was living homeless in the area, injected heroin, and had tested HIV-positive in June (patient 5). A social media search performed by a public health disease intervention specialist linked her to a man who injected drugs and was living homeless who had tested HIV-positive in July (patient 7). PHSKC was aware of three other recently diagnosed cases of HIV infection among women who inject drugs and were living homeless in north Seattle (patients 1, 2, and 3); none of these women had known epidemiologic links to other recently diagnosed cases. Subsequent molecular analyses conducted with HIV TRACE (*2*), a program that uses HIV genotypes to identify cases with related HIV strains based on HIV genetic sequence data, confirmed that four of the recently diagnosed cases in women and MSW who inject drugs, including the three without known epidemiologic links to other 2018 diagnoses, were infected with related HIV strains (patients 1–4). Molecular analysis also linked the seven recently diagnosed cases to eight cases diagnosed during 2008–2017 (patients 15–21 and 23) and two cases identified in September 2018 (patients 11 and 12). As of November 20, 2018, the cluster included 23 cases (Figure) (Table 2), 14 of which were diagnosed in 2018, demonstrating that transmission was at least intermittently ongoing since 2008, with evidence suggesting an acceleration in transmission during 2017–2018.

**TABLE 1:** Clinical and epidemiologic characteristics of a cluster of human immunodeficiency virus (HIV) cases among 23 persons living homeless who inject drugs and their sex partners and molecularly linked cases — Seattle, Washington, 2008–2018

Patient no.	Diagnosis quarter/yr	Gender	HIV risk factor and substance use	Reported exchange of sex	Reason for HIV testing	Date last HIV test*	Links to other cases identified through investigation	Related HIV strain	Cluster criteria^†^	Care status^§^
**HIV infection diagnosed 2018**										
1	Q1, 2018	F	Heroin/meth (IDU)	No	Regular testing	Q4, 2013	None	Yes	2,3	Suppressed
2	Q1, 2018	F	Heroin/meth (IDU uncertain)	No	STD symptoms	Unknown	Sex	Yes	1,2,3	In care, not suppressed
3	Q1, 2018	F	Heroin/meth (IDU)	No	Acute HIV symptoms	Never tested	None	Yes	2,3	Suppressed
4	Q2, 2018	M	Heroin (IDU); meth (smoke)	No	Hospitalized	Q1, 2017	IDU	Yes	1,2,3	In care, not suppressed
5	Q2, 2018	F	Heroin (IDU); meth (smoke)	Yes	Court-ordered testing	Unknown	Sex; IDU	ND	1,2	Out of care
6^¶^	Q3, 2018	M	NIR: presumed heterosexual; heroin, meth (non-IDU)	Yes	Acute HIV symptoms	Unknown	Sex	ND	1,2	In care, not suppressed
7	Q3, 2018	M	Heroin/meth (IDU)	No	Surveillance outreach testing	Unknown	Social media; IDU	ND	1,2	Out of care
8	Q3, 2018	F	Heroin (IDU); meth (smoke)	Yes	Outreach	Q4, 2017	None	ND	2	Out of care
9	Q3, 2018	F	Heroin (IDU); meth (smoke)	Yes	Outreach	Q4, 2016	None	ND	2	Suppressed
10	Q3, 2018	F	Unknown drugs (IDU)	Yes	Acute HIV symptoms	Unknown	No interview	ND	2	Out of care
11	Q3, 2018	F	Meth (IDU)	Yes	ED screening	Q1, 2018	No interview	Yes	2,3	In care, not suppressed
12	Q3, 2018	F	Heterosexual	Yes	Outreach	Q3, 2018	Sex	Yes	2,3	Suppressed
13	Q4, 2018	F	Heroin (IDU); meth (unknown route)	Yes	Mobile clinic	Q4, 2013	No interview	ND	2	In care, not suppressed
14	Q4, 2018	F	Meth (IDU)	Yes	Outreach	Q1, 2018	No interview	ND	2	Out of care
**HIV infection diagnosed 2008–2017**										
15	Q1, 2008	F	Heterosexual	Unknown	Unknown	Q1, 2006	No interview	Yes	3	Deceased
16	Q2, 2008	M	Unknown drugs (IDU)	Unknown	Unknown	Unknown	None	Yes	3	Deceased
17	Q3, 2011	M	NIR; Unknown drug use	Unknown	Unknown	2009	Sex	Yes	1,3	Out of care
18	Q3, 2014	F	NIR; history of IDU (none recently)	No	Acute HIV symptoms	2000	None	Yes	3	In care, not suppressed
19	Q4, 2016	F	Heroin (IDU); crack cocaine	Yes	HIV-unrelated infection	Q4, 2008	None	Yes	3	Suppressed
20	Q4, 2016	M	Heroin/meth (IDU)	Unknown	Unknown	2014	No interview	Yes	3	Suppressed
21	Q4, 2016	F	Unknown drugs (IDU)	Unknown	Unknown	Unknown	Sex	Yes	1,3	Suppressed
22	Q2, 2017	M	Heterosexual; unknown drug use	Unknown	Unknown	Unknown	Sex	ND	1	Out of care
23	Q4, 2017	F	Heroin (IDU); meth (unknown route)	No	Regular testing	Unknown	None	Yes	3	Suppressed

**Abbreviations:** ED = emergency department; F = female; IDU = injection drug use; M = male; Meth = methamphetamine; MSW = men who have sex with women; ND = no data available; NIR = no identified risk; Q = quarter.

* Most recent test based on patient self-report or verified result from medical record. Quarter not specified when unknown.

^†^ Cluster criteria: 1 = HIV infection diagnosis in a woman or MSW in 2018, with partner services data indicating sex or sharing injection-drug equipment with a previously identified cluster case; 2 = HIV infection diagnosis in 2018 in a woman or MSW living homeless in the outbreak area; 3 = molecular analysis indicating HIV infection with a strain related to those identified among persons meeting either of the first two criteria (HIV-TRACE genetic distance ≤1.5%). Cases were excluded if molecular analysis indicated infection with an HIV strain unrelated to the cluster.

^§^ Suppression (<200 copies of HIV RNA/mL of blood) based on most recent HIV RNA test result performed during September 1, 2017–September 17, 2018.

^¶^ Index case for cluster.

**FIGURE:**
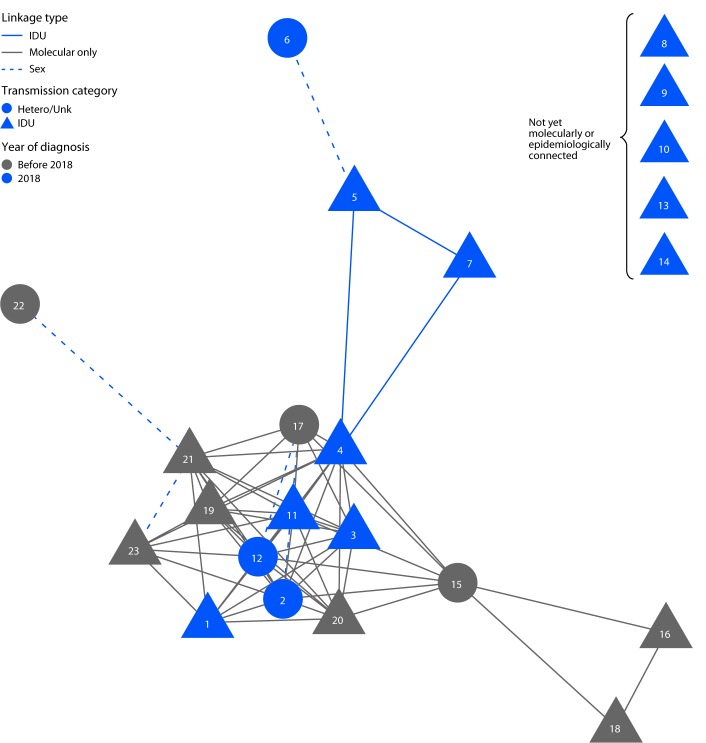
Human immunodeficiency virus (HIV) transmission network among heterosexual men and women who inject drugs,* by linkage type,^†^ transmission category, and year of diagnosis — Seattle, Washington, 2008–2018 **Abbreviations:** Hetero/Unk = heterosexual/unknown; IDU = injection drug use. * N = 23; includes sex partners of persons who inject drugs and those with a molecularly linked HIV strain. ^†^ Molecular linkages do not necessarily indicate a direct epidemiologic connection between two cases, and line lengths are not reflective of the degree of relatedness of each molecular linkage.

**TABLE 2:** Demographic and behavioral characteristics of 23 persons living homeless who inject drugs and their sex partners and molecularly linked cases in a cluster of human immunodeficiency virus (HIV) transmission — Seattle, Washington, 2008–2018

Characteristic	No. (%)	
	2018 cases (n = 14)	All cases (n = 23)
**Median age (range) (yrs)**	39 (22–61)	39 (21–65)
**Race/Ethnicity**		
White, non-Hispanic	11 (78)	17 (74)
Black, non-Hispanic	2 (14)	2 (9)
Latino	0 (—)	2 (9)
Multiracial	1 (7)	2 (9)
**Gender**		
Female	11 (79)	16 (70)
Male	3 (21)	7 (30)
**HIV risk factor**		
Injection drug use	12 (86)	16 (70)
Heterosexual	1 (7)	3 (13)
No identified risk	1 (7)	3 (13)
**Drug use**		
Heroin and methamphetamine	10 (71)	12 (52)
Heroin without methamphetamine	0 (—)	1 (4)
Methamphetamine without heroin	2 (14)	2 (8)
None	1 (7)	3 (13)
Injection drug use of unknown drug	1 (7)	3 (13)
Unknown	0 (—)	2 (9)
Women who exchange sex*	**9 (82)**	**10 (73)**

* Includes data from all 11 women with diagnoses in 2018 and 14 of 16 women in the entire cluster.

All 14 cases diagnosed in 2018 occurred in persons living homeless in an area of approximately 3 square-miles; 11 were in women who identified as cisgender, nine of whom reported exchanging sex for money or nonmonetary items, and 12 were in persons who inject drugs, 10 of whom used both heroin and methamphetamine.

Analysis of all newly reported HIV infections during January 1–November 30, 2018, identified 27 cases of HIV infection among women and MSW who inject drugs in King County. This represents a 286% increase over the seven cases diagnosed in 2017. 

## Public Health Response

On August 3, 2018, a PHSKC disease intervention specialist identified epidemiologic links among patients 5, 6, and 7. Four days later, the health department issued an alert to medical and social service providers concerning the cluster and the increase in HIV diagnoses among persons who inject drugs and who are living homeless. The HIV/Sexually Transmitted Diseases program also contacted several local EDs and the hospital closest to where the patients lived. These EDs have asked providers to increase screening of persons who inject drugs and persons who are living homeless, and at least three are developing more systematic, risk-based opt-out HIV screening programs. To date, ED screening has identified one case of HIV infection (patient 11). On August 20, the King County Jail expanded HIV testing, including opt-out testing at health assessments at 10–14 days and, when resources permit, at time of jail booking. This effort has identified one new case of HIV infection, which has not been linked to the cluster. PHSKC also initiated an expanded program of outreach testing, condom distribution, and syringe services among persons living homeless in north Seattle. As of November 15, 2018, that initiative had tested 534 persons and identified four related cases of HIV infection (patients 8, 9, 12, and 14).

PHSKC is increasing access to HIV testing and preexposure prophylaxis (PrEP) among persons who inject drugs through its sexually transmitted disease clinic and SSPs and via a collaboration with a mobile clinic serving north Seattle women who exchange sex or are living homeless. PHSKC is also conducting a rapid assessment to define the medical and social service needs and preferences of persons who inject drugs or who are living homeless in north Seattle with the goal of expanding services, including medication-assisted treatment. Investigations of this cluster and efforts to link infected persons to care are ongoing.

## Discussion

This report describes an outbreak of HIV infection in a population of women and MSW who inject drugs and the sex partners of these persons. The outbreak was part of a cluster of 23 persons, nine of whom received a diagnosis of HIV during 2008‒2017. The data suggest that HIV transmission from persons with these earlier diagnoses, some of whom were not virally suppressed, or from their sex partners without a diagnosis, led to a rapid expansion of transmission during 2017–2018, with 14 related infections diagnosed in 2018 in a small geographic area.

The occurrence of a large HIV outbreak in Indiana in 2014–2015 (*1*) highlighted the vulnerability of rural communities with few HIV prevention and medical services to HIV outbreaks among persons who inject drugs. Subsequent CDC analyses sought to identify the 5% of U.S. counties with the highest risk for HIV and hepatitis C virus outbreaks among persons who inject drugs (*3*). King County, Washington, was not among those highest-risk counties. PHSKC estimates that 93% of county residents with HIV infection know their HIV status and that 85% of persons with diagnosed infection were virally suppressed in 2017 (<200 copies of HIV RNA/mL of blood) (*4*). The rate of new diagnoses of HIV infection in King County declined 51% from 2008 to 2017 (PHSKC, unpublished data, 2019). PHSKC SSPs provided >7 million syringes to persons who inject drugs in 2017; 79% of persons who inject drugs report using SSPs, and syringe sharing among persons who inject drugs has declined over time (*5*). Only 1%–3% of the approximately 21,000 women and MSW who inject drugs in the county have HIV infection, and 80% of those with a diagnosis are virally suppressed (*4*). Despite these successes, the current outbreak, similar to a recent outbreak in Massachusetts, demonstrates that vulnerability to outbreaks of HIV infection among persons who inject drugs is widespread in the United States (*6*).

The outbreak described here is part of a larger increase in HIV infection among heterosexual persons who inject drugs that is ongoing in King County. During 2018, the county experienced a nearly threefold increase in new HIV infections among women and MSW who inject drugs. Several factors might contribute to King County’s vulnerability. First, although access to HIV care and prevention in the county is generally good, this outbreak was concentrated in an area where syringe and clinical services for persons who inject drugs are limited, highlighting the need to expand access. Second, like much of the United States, King County faces growing epidemics of opioid overdose and homelessness. From 2007 to 2018, the number of heroin overdose deaths in the county increased 264% (*7*), and from 2007 to 2017, the number of country residents living homeless increased 47% (*8*). Among SSP users surveyed in 2017, 43% were living homeless, and an additional 26% were unstably housed, a 19% increase from 2015 (*4*). Thus, the area has a rapidly growing population who inject drugs and are living homeless, a group for whom accessing services is particularly difficult. These factors have resulted in a new population-level susceptibility to HIV transmission.

The King County outbreak also illustrates both the value and limitations of disease intervention specialist investigations and molecular HIV analyses. Disease intervention specialists initially identified the outbreak, and PHSKC and the Washington State Department of Health used molecular analyses to recognize related cases not identified through disease investigation and to confirm relationships suggested by epidemiologic linkages. Retrospective review of the molecular data demonstrated that 10 related cases (eight with genetic sequence data available) were diagnosed from December 2016 to August 2018, when the cluster was first identified. Had the molecular data been available and analyzed more quickly, it might have been possible to respond earlier, possibly averting some cases. CDC recently initiated a national effort to expand the use of molecular HIV analyses to identify growing clusters of cases (*9*). The experience described here suggests how such analyses might be useful if they were available and analyzed in real time with appropriate thresholds for action.

Finally, the King County outbreak demonstrates how difficult it is to engage the most socially marginalized persons with medical care. As of mid-November 2018, seven of the 21 living persons in the cluster were not receiving HIV care. Disease intervention specialists are actively seeking these persons to link them to a clinic that provides walk-in HIV medical care (*10*).

Persons who inject drugs remain vulnerable to outbreaks of HIV infection, even in cities with large HIV prevention programs and shrinking HIV epidemics. A new U.S. Department of Health and Human Services initiative, Ending the HIV Epidemic: A Plan for America,* defines molecular HIV surveillance and associated responses as one of four central pillars for ending the epidemic. The outbreak described in this report illustrates the benefits of integrating disease investigations and molecular HIV analyses to more rapidly and efficiently identify and respond to localized outbreaks of HIV infection and should prompt health departments in other jurisdictions to investigate whether similar outbreaks are ongoing in their areas.

**Summary**

**What is already known about this topic?**

Although diagnoses of human immunodeficiency virus (HIV) infection among persons who inject drugs in the United States are declining, an HIV outbreak among such persons in rural Indiana demonstrated that population’s vulnerability to HIV infection.

**What is added by this report?**

In 2018, disease investigation and molecular HIV surveillance in Seattle, Washington, identified 14 related HIV diagnoses among heterosexuals who were living homeless, most of whom injected drugs. From 2017 to mid-November 2018, the number of HIV diagnoses among heterosexuals in King County, Washington, who inject drugs increased 286%.

**What are the implications for public health practice?**

Persons who inject drugs, particularly those living homeless, remain vulnerable to outbreaks of HIV infection, even in cities with large HIV prevention programs and shrinking HIV epidemics.
